# Basic Conditioning Factors' Influences on Adolescents' Healthy Behaviors, Self-Efficacy, and Self-Care

**DOI:** 10.1080/01460860601087156

**Published:** 2006-12-12

**Authors:** Donna Callaghan

**Affiliations:** Widener University, School of Nursing, Chester, Pennsylvania

## Abstract

This article reports a secondary statistical analysis of data from a study investigating the relationships among health-promoting self-care behaviors, self-care self-efficacy, and self-care agency in an adolescent population (Callaghan, 2005). The purpose of this study was to identify the influences of selected basic conditioning factors on the practice of healthy behaviors, self-efficacy beliefs, and ability for self-care in 256 adolescents. The research instruments used to collect data for this study include: Health-Promoting Lifestyle Profile II Scale; Self-Rated Abilities for Health Practices Scale; Exercise of Self-Care Agency Scale; demographic questionnaire assessing basic conditioning factors. The results of this analysis identified significant relationships between the following basic conditioning factors and adolescents' practice of healthy behaviors, self-efficacy of those behaviors, and self-care abilities: support system, adequate income, adequate living conditions, gender, routine practice of religion, and reported medical problems/disabilities. These findings can give adolescent health nurses direction in developing interventions that promote the self-care and health in this specific population.

A secondary statistical analysis of data from a study investigating the relationships among health-promoting self-care behaviors, self-care self-efficacy, and self-care agency in an adolescent population (Callaghan, 2005) is reported in this article. Pender's Health Promotion Model (2002), Bandura's Self-Efficacy Theory (1997), and Orem's Self-Care Deficit Nursing Theory (2001) were used in the development of the conceptual framework for this study. The study concepts were measured by three instruments based on these theories/models that included the Health-Promoting Lifestyle Profile II scale, the Self-Rated Abilities for Health Practices scale, and the Exercise of Self-Care Agency scale.

A demographic questionnaire developed by this researcher was used to assess selected basic conditioning factors and was analyzed in relation to the adolescents' practice of healthy behaviors, self-efficacy beliefs, and ability for self-care. Orem (2001) identifies 10 basic conditioning factors in the conceptualization of self-care agency, including age, gender, developmental state, health state, pattern of living, health care system factors, family system factors, sociocultural factors, availability of resources, and external environmental factors. The demographic questionnaire used in this study included items that assessed age, gender, grade, family structure, number of siblings, support system, education of parents, employment of self and parents, race, practice of religion, adequacy of income, adequacy of health insurance, adequacy of living conditions, and medical problems/disabilities. These questions were developed based on a report by Moore & Pichler (2000), who found the least measured basic conditioning factors to be pattern of living, resource availability, developmental state, environmental state, and healthcare system factors. The statistically significant relationships between these basic conditioning factors and the concepts of health-promoting self-care behaviors, self-care self-efficacy, and self-care agency are reported in this study.

## REVIEW OF THE LITERATURE

Studies that measure basic conditioning factors' influences on adolescent self-care agency and self-care practices were reported in nursing literature. Canty-Mitchell (2001) identified a significant positive correlation between hope and self-care agency (*r* = .47, *p* < .001). McCaleb and Cull (2000) identified a relationship between general self-care practices and church attendance (*r* = . 20, *p* = .0001). Renker (1999) identified an interaction effect of abuse and the social support factors of shelter and family help on infant birth weights of adolescent mothers. Mosher and Moore (1998) identified a significant relationship between self-concept and self-care practices (*r* = .23, *p* ≤ .05). Self-esteem was identified as a component of an adolescent's self-care agency through a concept analyses and was found to be influenced by basic conditioning factors (Anderson & Olnhausen, 1999). In this study the relationships of selected basic conditioning factors with adolescents' health-promoting self-care behaviors, self-care self-efficacy, and self-care agency are investigated.

## RESEARCH QUESTION

The following research question was investigated in this study: What are the relationships between selected basic conditioning factors and health-promoting self-care behaviors, self-care self-efficacy, and self-care agency in an adolescent population?

## CONCEPTUAL FRAMEWORK

The conceptual framework for this study was an integration of three concepts from Pender's Health Promotion Model (2002), Bandura's Social Cognitive Theory (1997), and Orem's Self-Care Deficit Nursing Theory (2001). The following quote describes this conceptual integration:

Self-care agency is a broad concept that includes three capabilities: foundational capabilities, power components, and self-care operations. The transitional capability of self-care operations involves the judgment of one's ability for self-care that is consistent with the conceptualization of self-care self-efficacy. Self-care self-efficacy involves judgment of one's ability to perform self-care behaviors. The productive capability of self-care operations involves the actual performance of self-care behaviors. These behaviors are learned and can be directed towards the performance of specific behaviors that can lead to the promotion of health. These learned behaviors can be conceptualized as health-promoting self-care behaviors (Callaghan, 2003, p. 248).

## METHODOLOGY

Descriptive and inferential statistics were used to identify the relationships between basic conditioning factors and health-promoting self-care behaviors, self-care self-efficacy, and self-care agency in adolescents. The basis for nursing interventions that promote adolescent self-care and health could be established by the results of this analysis.

### Procedure

After approvals were obtained from the university IRB and the high school administration, the convenience sample of adolescents aged 14 to 19 were recruited from a Southern New Jersey High School. The school was chosen because of the diversity of the student body. Adolescents in the high school's health classes were invited to participate in the study. A power analysis revealed that at least 235 subjects were needed to attain a power = .99; medium effect size (f^2^ = .15); and *p* = .05. A total of 550 explanation of study forms, assent forms, and consent forms were distributed by the health teachers to these students for signatures. The researcher was given one school day to administer the questionnaires to each of the high school health classes. On this day, 265 consent forms signed by the students' guardians as well as 265 assent forms signed by the students were obtained. These 265 students received the questionnaires to complete in the 30-minute health class period. Of those 265 questionnaires distributed to the students, 262 were returned to the researcher. Six of these questionnaires were unusable because of missing data, which resulted in a 47% response rate.

### Sample

The data obtained from the demographic questionnaire describing the convenience sample obtained for this study are presented in Table 1. The demographic questionnaire included nominal, ordinal, interval, and ratio scales. The measures of adequate income, health insurance, and living conditions were obtained through items requiring a “yes” or “no” response—for example, “Do you feel that your family has an adequate income to meet your daily needs?”

**Table 1:** Demographic statistics (N = 256)

Characteristic	n	%
Age 14	18	7
15	56	22
16	73	28
17	57	22
18	45	18
19	7	3
Grade 9	65	25
10	69	27
11	55	22
12	66	26
Female	149	58
Lived with both parents	182	71
Had siblings	239	95
Had a support system	242	95
Mother high school grad	240	95
Father high school grad	249	94
Student not employed	139	55
Mother employed full-time	151	59
Father employed full-time	232	94
White race	190	74
Routinely practiced of religion	28	50
Had adequate income	235	92
Had adequate health insurance	246	97
Had adequate living conditions	248	98
Had no medical problems	196	77

### Instruments

The four surveys distributed to the students included the Health-Promoting Lifestyle Profile II (HPLPII), the Self-Rated Abilities for Health Practices (SRAHP), the Exercise of Self-Care Agency (ESCA), and a demographic questionnaire. The HPLPII (Walker, Sechrist, & Pender, 1987), which assesses how frequently health-promoting behaviors are practiced, was used to measure the variable of health-promoting self-care behaviors. The HPLPII is a 52-item 4-point Likert-type scale that consists of six sub-scales: health responsibility, physical activity, nutrition, interpersonal relations, spiritual growth, and stress management. Adequate Cronbach's alpha coefficients of internal consistency reliability were reported in a study of 379 adults with coefficients ranging from .75 to 93 (Callaghan, 2003). In this study the Cronbach's alpha coefficients of internal consistency reliability were total scale .92, health responsibility .81 (9 items), physical activity .79 (8 items), nutrition .71 (9 items), spiritual growth .82 (9 items), interpersonal relations .82 (9 items), and stress management .64 (8 items).

The SRAHP (Becker, Stuifbergen, Soo Oh, & Hall, 1993) consists of a 28-item 5-point Likert-type scale that assesses the confidence one has in abilities to perform self-care behaviors. This scale was used to measure the variable of self-care self-efficacy in four areas: exercise, psychological well-being, nutrition, and health practices. Adequate Cronbach's alpha coefficients of internal consistency reliability were reported in a study of 379 adults with coefficients ranging from .81 to 94 (Callaghan, 2003). In this study the Cronbach's alpha coefficients were total scale .93 (7 items), nutrition .82 (7 items), psychological well-being .81 (7 items), exercise .88 (7 items), and responsible health practices .81 (7 items).

The ESCA (Kearney & Fleischer, 1979), revised according to construct and discriminant validity findings reported by Reisch and Hauck (1988), was used to measure the variable of self-care agency. The questions in this scale assess one's ability for self-care including the areas of self-concept, initiative and self-responsibility, knowledge and information seeking, and passivity. Adequate Cronbach's alpha coefficients of internal consistency reliability were reported in a study of 379 adults with coefficients ranging from .76 to 91 (Callaghan, 2003). In this study the Cronbach's alphas were total scale .89, self-concept .83 (12 items), initiative and responsibility .82 (12 items), knowledge and information-seeking .75 (5 items), and passivity .62 (6 items). Since the subscale of passivity is below .70, any significant results related to this variable should be interpreted with caution.

A demographic questionnaire assessing basic conditioning factors of self-care agency was developed by this researcher for use in the adolescent population in this study. The instrument included questions that assessed age, gender, grade, family structure, number of siblings, support system, education of parents, employment of self and parents, race, practice of religion, perception of adequacy of income, perception of adequacy of health insurance, perception of adequacy of living conditions and medical problems/disabilities. The specific questions assessing perceptions were measured through dichotomous “yes” or “no” responses.

### Data Analysis

Statistical analyses, including Pearson's correlations, Independent t-tests, and Analysis of Variance (ANOVA), were computed on the data using version 10.0 of the Statistical Package for the Social Sciences (SPSS). Pear-son's correlations did not identify any significant relationships between age and the study variables of health-promoting self-care behaviors, self-care self-efficacy, and self-care agency. Analysis of Variance did not identify any significant differences among groups defined by grade level, family structure, education and employment status of mother and father, and medical problems related to the study variables. No significant differences were found among study variables when students' current employment and perception of adequacy of health insurance were analyzed by t-tests.

Significant t-tests were identified relative to the basic conditioning factors of support system, adequate income, adequate living conditions, gender, routine practice of religion, and reported medical problems exhibited. Those students who were female, responded “yes” to the questions related to presence of a support system, adequate income, adequate living conditions, routine practice of religion, and medical problems had higher mean scores on many of the total scale and subscale scores of the variable measurements. Levine's tests for equality of variance were nonsignificant for the results reported in this study.

## RESULTS

The most significant finding in this study was the influence of a support system on an adolescent's healthy behaviors, self-efficacy, and self-care. Adolescents who indicated “yes” to the question “Do you have a support system (family, friends, teachers, neighbors, healthcare providers, clergy) whom you feel free to ask for help when needed?” also reported higher mean scores on 13 of the 14 variables measured in this study. These variables include the six subscales of the measure of health-promoting self-care behaviors (the routine practice of health responsibility, physical activity, nutrition, interpersonal relations, spiritual growth, and stress management), the six subscales of self-care self-efficacy (self-efficacy of exercise, psychological well-being, nutrition, and health practices), and three of the four subscales of self-care agency (self-concept, initiative and self-responsibility, and knowledge and information seeking). There was no difference between groups relative to the variable of passivity, which is the fourth subscale in the measurement of self-care agency. Therefore, those high school students having support systems more often practiced healthy behaviors, had high levels of self-efficacy related to the practice of these behaviors, and had more abilities for self-care. These findings are presented in Table 2.

**Table 2:** Independent t-tests on variable of support system (N = 254)

Scale	Support System	M (SD)	t	p
*HPLPII: Total Scale*				
	Yes	133.0 (21.2)	4.76	.00
	No	103.5 (14.1)		
Health Responsibility				
	Yes	18.0 (4.7)	4.00	.00
	No	12.5 (3.4)		
Nutrition				
	Yes	20.95 (4.7)	2.10	.04
	No	18.0 (4.6)		
Spiritual Growth				
	Yes	27.00 (5.03)	3.26	.00
	No	22.17 (4.76)		
Interpersonal Relationships				
	Yes	26.99 (4.9)		
	No	19.4 (3.8)	5.28	.00
Stress Management				
	Yes	20.0 (3.9)	3.34	.00
	No	16.1 (4.8)		
*SRAHP: Total Scale*				
	Yes	73.5 (19.1)	5.75	.00
	No	41.3 (14.6)		
Nutrition				
	Yes	16.9 (5.9)	3.56	.00
	No	10.7 (3.96)		
Psychological Well-Being				
	Yes	18.09 (5.59)	5.49	.00
	No	9.08 (4.38)		
Exercise				
	Yes	18.9 (6.9)	4.10	.00
	No	10.6 (4.5)		
Health Responsibility				
	Yes	19.6 (5.5)	5.35	.00
	No	11.0 (5.3)		
*ESCA: Total Scale*				
	Yes	90.5 (19.02)	3.63	.00
	No	70.2 (17.74)		
Self-Concept				
	Yes	36.1 (7.5)	3.57	.00
	No	28.1 (8.2)		
Initiative and Responsibility				
	Yes	31.66 (5.13)	4.23	.00
	No	25.25 (4.94)		
Knowledge & Information-Seeking				
	Yes	12.3 (4.2)	3.45	.00
	No	8.0 (3.2)		

Students who responded “yes” to the question “Do you feel that your family has an adequate income to meet your daily needs” also reported higher mean scores on the total scales of all three instruments and the HPLPII subscales of spiritual growth, interpersonal relations, and stress management, the SRAHP subscales of nutrition and exercise, and the ESCA subscales of self-concept, initiative and self-responsibility, and knowledge and information-seeking. These results indicate that high school students who perceive that they have the monetary resources to meet daily needs also practice healthy behaviors more frequently, have higher self-care self-efficacy levels, and have more self-care abilities. These results are presented in Table 3.

**Table 3:** Independent t-tests on variable of adequate income (N = 255)

Scale	Adequate Income	M (SD)	t	p
*HPLPII: Total Scale*				
	Yes	132.97 (21.0)	3.51	.00
	No	115.6 (24.1)		
Spiritual Growth				
	Yes	27.0 (4.9)	2.84	.01
	No	23.7 (6.7)		
Interpersonal Relations				
	Yes	26.9	4.90	.00
	No	22.8		
Stress Management				
	Yes	20.0 (3.9)	2.79	.01
	No	17.5 (4.9)		
*SRAHP: Total Scale*				
	Yes	73.5 (18.98)	4.21	.00
	No	54.6 (23.4)		
Nutrition				
	Yes	17.0 (5.8)	4.12	.00
	No	11.5 (5.4)		
Exercise				
	Yes	18.9 (6.8)	2.48	.01
	No	14.9 (8.12)		
*ESCA: Total Scale*				
	Yes	90.7 (18.7)	3.15	.00
	No	76.7 (22.39)		
Self-Concept				
	Yes	36.2 (7.4)	3.80	.00
	No	29.5 (9.3)		
Initiative and Responsibility				
	Yes	31.7 (5.1)	3.42	.00
	No	27.6 (6.5)		
Knowledge & Information-Seeking				
	Yes	12.2 (4.2)	2.17	.03
	No	10.1 (4.6)		

Independent t-tests performed on the data obtained from the question “Do you feel that your family's living conditions are adequate to maintain your health and well-being?” indicated a difference between students answering “yes” and “no” to this question. Those who responded affirmatively reported higher mean scores on the total scales of the HPLPII and the ESCA. These students also reported higher mean scores on the HPLPII subscales of health responsibility, nutrition, and stress management, the SRAHP subscale of psychological well-being, and the ESCA subscales of self-concept and initiative and self-responsibility. This finding indicates that high school students who perceived that they had adequate living conditions also practice healthy behaviors more frequently and have higher self-efficacy levels regarding the practice of healthy behaviors. These results are listed in Table 4.

**Table 4:** Independent t-tests on variable of adequate living conditions (N = 254)

Scale	Adequate Living Conditions	M (SD)	t	p
*HPLPII: Total Scale*				
	Yes	132.1 (21.4)	2.67	.01
	No	108.3 (30.1)		
Health Responsibility				
	Yes	17.8 (4.7)	2.22	.03
	No	13.5 (4.7)		
Nutrition				
	Yes	20.9 (4.7)	2.61	.01
	No	15.8 (6.1)		
Stress Management				
	Yes	19.9 (3.96)	2.38	.02
	No	16.0 (5.9)		
*SRAHP*				
Psychological Well-Being				
	Yes	17.8 (5.7)	2.15	.03
	No	12.7 (6.9)		
*ESCA: Total Scale*				
	Yes	90.1 (19.0)	2.47	.01
	No	70.5 (24.9)		
Self-Concept				
	Yes	35.8 (7.6)	2.03	.04
	No	29.3 (11.2)		
Initiative and Responsibility				
	Yes	31.4 (5.2)	2.19	.03
	No	26.7 (7.1)		

In relation to gender, females had higher mean scores on the SRAHP total scale and subscales of nutrition and health responsibility, the HPLPII subscales of health responsibility and interpersonal relations, and the ESCA subscale of knowledge and information-seeking. This finding indicates that females had more confidence in abilities to practice healthy behaviors than males in this sample. These results are reported in Table 5.

**Table 5:** Independent t-tests on variable of gender (N = 256)

Scale	Gender	M (SD)	t	p
*HPLPII*				
Health Responsibility				
	M	16.5 (4.4)	−3.76	.00
	F	18.69 (4.9)		
Interpersonal Relations				
	M	25.3 (4.7)	−3.45	.00
	F	27.5 (5.2)		
*SRAHP: Total Scale*				
	M	68.8 (20.4)	−2.13	.03
	F	74.2 (19.4)		
Nutrition				
	M	14.9 (6.2)	−3.98	.00
	F	17.8 (5.6)		
Health Responsibility				
	M	17.9 (5.7)	−3.09	.00
	F	20.1 (5.6)		
*ESCA*				
Knowledge and Information-Seeking				
	M	11.0 (4.0)	−3.33	.00
	F	12.8 (4.3)		

Independent t-tests performed on the data obtained from the question “Do you routinely practice a religion?” indicated students answering “yes” had higher mean scores on the total scales of the HPLPII and SRAHP, the HPLPII subscale of nutrition, the SRAHP subscale of nutrition, and ESCA subscale of initiative and responsibility. This finding indicates the students who routinely practiced a religion also practiced health-promoting self-care behaviors more frequently and had higher self-care self-efficacy levels. Table 6 presents these results.

**Table 6:** Independent t-tests on variable of routine practice of religion (N = 254)

Scale	Routine Practice of Religion	M (SD)	t	p
*HPLPII: Total Scale*				
	Yes	134.95 (21.0)	2.53	.01
	No	128.12(22.10		
Nutrition				
	Yes	21.9 (4.7)	3.61	.00
	No	19.8 (4.6)		
*SRAHP: Total Scale*				
	Yes	75.2 (18.3)	2.62	.01
	No	68.7 (21.2)		
Nutrition				
	Yes	17.6 (5.9)	2.69	.01
	No	15.6 (5.9)		
*ESCA*				
Initiative and Responsibility				
	Yes	32.11 (5.13)	2.41	.02
	No	30.53 (5.37)		

The students who reported having medical problems or disabilities also reported higher scores on the SRAHP total and subscales of nutrition, exercise, and health responsibility and the HPLPII subscale of health responsibility. Therefore, high school students who have medical problems or disabilities indicated higher levels of self-efficacy related to the practice of healthy behaviors and were more responsible for health in general. These findings are presented in Table 7.

**Table 7:** Independent t-tests on variable of medical problems/disabilities (N = 256)

Scale	Medical Problems/Disabilities	M (SD)	t	p
HPLPII				
Health Responsibility				
	Yes	18.93 (5.23)	2.18	.03
	No	17.40 (4.60)		
SRAHP: Total Scale				
	Yes	77.28 (17.86)	2.38	.02
	No	70.32 (20.36)		
Nutrition				
	Yes	18.32 (5.86)	2.62	.01
	No	16.03 (5.94)		
Exercise				
	Yes	20.63 (6.47)	2.71	.01
	No	17.85 (7.09)		
Health Responsibility				
	Yes	20.58 (5.25)	2.12	.04
	No	18.80 (5.82)		

## DISCUSSION

Through a secondary analysis of data collected from Callaghan's study (2005), relationships were identified between the following basic conditioning factors and health-promoting self-care behaviors, self-care self-efficacy, and self-care agency: perception of support system, perception of adequate income, perception of adequate living conditions, gender, routine practice of religion, and reported medical problems. Considering that the generalizability of this study is limited to similar adolescent populations, these findings can give adolescent health nurses, specifically school nurses, direction for health-promotion and self-care interventions. Since perception of adequate income and living conditions are related to the practice of healthy behaviors, self-efficacy, and self-care, school nurses can assess the demographics of the student body in order to identify those with deficits meeting these basic needs. Knowledge of social support systems within the community that can assist high school students as well as families in meeting these basic needs is of importance in the practice of school nursing. Meeting these basic needs is the first step in promoting health and self-care in this high school population.

The influence of gender and religion on an adolescent's health and self-care also is of concern to school nurses. Again, knowledge of and access to social support systems within the community, such as faith community nurses, can be instrumental in assisting adolescents to identify and meet gender-specific health and self-care needs including spiritual health. The influence of having medical problems or disabilities on an adoles-cent's health and self-care may indicate that these students do receive social support from family, school nurses, and other health professionals in relation to these deficits, leading to higher self-care self-efficacy levels in this population.

A major significant finding was the relationship of the basic conditioning factor “support system” with the study variables. High school students who reported having a support system also reported practicing healthier behaviors, had higher levels of self-efficacy in relation to practicing these behaviors, and had more abilities for self-care than those who did not have a support system. The influence of a support system on high school students' practice of healthy behaviors has been identified in the nursing literature (Yarcheski et al., 2004).

Only one study addressing adolescent social support used Orem's Self-Care Deficit Nursing Theory as a theoretical framework. In this study the social support factors of shelter and family were identified as significant predictors of infant birth weights in older adolescent mothers (Renker, 1999). No studies that investigated the effects of social support interventions on healthy behaviors were identified in the nursing literature. Pender, Murdaugh, and Parsons (2002) provide directions for social support research including theory and intervention development that is culturally sensitive and across the life span. Interventions to promote social support are presented by Pender et al. (2002) as well. Since the family is the primary support group, the school nurse must identify and assess a high school student's family level of social support. Developing health promotion programs that involve the student as well as the family is imperative. Other support groups that can be utilized include community organizations such as churches. Through collaborating with churches, the school nurse could assist students in meeting basic needs as well as develop health promotion interventions for families in the community. The school nurse should have access to self-help group information to provide to the students and families since these groups also are an important source of support within most communities.

The high school nurse is one health care professional in a key position to promote the health and self-care of adolescents. One of the major implications of this study is the importance of social support for a teenager, specifically the influence social support has on the practice of healthy behaviors, self-efficacy of these behaviors, and abilities for self-care. Through the assessment of the adolescent's family support as well as the availability and use of other social support systems within the community, the high school nurse can identify the strengths and deficits within that support system. When deficits in social support are identified, the high school nurse can intervene through referring to or establishing these supports for the adolescent and the adolescent's family. A collaborative effort between the high school nurse and community organizations such as churches could assist in establishing this much needed social support in the lives of adolescents, possibly leading to a healthier teenage population.

## References

[b1] Anderson J. A., Olnhausen K. S. (1999). Adolescent self-esteem: A foundational disposition. Nursing Science Quarterly.

[b2] Bandura A. (1997). Self-efficacy: The exercise of control.

[b3] Becker H., Stuifbergen A., Soo Oh H., Hall S. (1993). Self-rated abilities for health practices: A health self-efficacy measure. Health Values.

[b4] Callaghan D. M. (2005). The influence of spiritual growth on adolescents' initiative and responsibility for self-care. Pediatric Nursing, 31.

[b5] Callaghan D. M. (2003). Health-promoting self-care behaviors, self-care self-efficacy, and self-care agency. Nursing Science Quarterly.

[b6] Canty-Mitchell J. (2001). Life change events, hope, and self-care agency in inner-city adolescents. Journal of Child and Adolescent Psychiatric Nursing.

[b7] Kearney B. Y., Fleischer B. J. (1979). Development of an instrument to measure exercise of self-care agency. Research in Nursing and Health.

[b8] McCaleb A., Cull V. V. (2000). Sociocultural influences and self-care practices of middle adolescents. Journal of Pediatric Nursing.

[b9] Moore J. B., Pichler V. H. (2000). Measurement of Orem's basic conditioning factors: A review of published research. Nursing Science Quarterly.

[b10] Mosher R. B., Moore J. B. (1998). The relationship of self-concept and self-care in children with cancer. Nursing Science Quarterly.

[b11] Orem D. E. (2001). Nursing: Concepts of practice.

[b12] Pender N. J., Murdaugh C. L., Parsons M. A. (2002). Health promotion in nursing practice.

[b13] Renker P. R. (1999). Physical abuse, social support, self-care, and pregnancy outcomes of older adolescents. JOGNN: Journal of Obstetric, Gynecologic, & Neonatal Nursing.

[b14] Riesch S. K., Hauck M. R. (1988). The exercise of self-care agency: An analysis of construct and discriminant validity. Research in Nursing & Health.

[b15] Walker S. N., Sechrist K. R., Pender N. J. (1987). The health-promoting lifestyle profile: Development and psychometric characteristics. Nursing Research.

[b16] Yarcheski A., Mahon N. E., Yarcheski T. J., Cannella B. L. (2004). A meta-analysis of predictors of positive health practices. Journal of Nursing Scholarship.

